# A deep-sea isopod that consumes *Sargassum* sinking from the ocean’s surface

**DOI:** 10.1098/rspb.2024.0823

**Published:** 2024-09-11

**Authors:** Logan M. Peoples, Mackenzie E. Gerringer, Johanna N. J. Weston, Rosa León-Zayas, Abisage Sekarore, Grace Sheehan, Matthew J. Church, Anna P. M. Michel, S. Adam Soule, Timothy M. Shank

**Affiliations:** ^1^ Flathead Lake Biological Station, University of Montana, Polson, MT, USA; ^2^ Department of Biology, State University of New York at Geneseo, Geneseo, NY, USA; ^3^ Biology Department, Woods Hole Oceanographic Institution, Woods Hole, MA, USA; ^4^ Biology Department, Willamette University, Salem, OR, USA; ^5^ Department of Applied Ocean Physics and Engineering, Woods Hole Oceanographic Institution, Woods Hole, MA, USA; ^6^ Graduate School of Oceanography, University of Rhode Island, Narragansett, RI, USA

**Keywords:** Munnopsidae, macroalgae, carbon sequestration, gut microbiome, deep sea, *Alvin*

## Abstract

Most deep-ocean life relies on organic carbon from the surface ocean. While settling primary production rapidly attenuates in the water column, pulses of organic material can be quickly transported to depth in the form of food falls. One example of fresh material that can reach great depths across the tropical Atlantic Ocean and Caribbean Sea is the pelagic macroalgae *Sargassum*. However, little is known about the deep-ocean organisms able to use this food source. Here, we encountered the isopod *Bathyopsurus nybelini* at depths 5002–6288 m in the Puerto Rico Trench and Mid-Cayman Spreading Center using the Deep Submergence Vehicle *Alvin*. In most of the 32 observations, the isopods carried fronds of *Sargassum*. Through an integrative suite of morphological, DNA sequencing, and microbiological approaches, we show that this species is adapted to feed on *Sargassum* by using a specialized swimming stroke, having serrated and grinding mouthparts, and containing a gut microbiome that provides a dietary contribution through the degradation of macroalgal polysaccharides and fixing nitrogen. The isopod’s physiological, morphological, and ecological adaptations demonstrate that vertical deposition of *Sargassum* is a direct trophic link between the surface and deep ocean and that some deep-sea organisms are poised to use this material.

## 1. Introduction

In the planet’s largest habitat—the deep sea—most life relies on fixed organic carbon and energy that originates in the surface ocean. Because only a small fraction of surface-derived primary production makes it to the deep seafloor, with most consumed by pelagic organisms throughout the water column (e.g. [1]), deep-ocean fauna are often energy and carbon limited. However, some organic material can sink quickly, bypassing pelagic processing and degradation. This rapidly transported carbon includes episodic deposits of microbial phytoplankton (e.g. [2,3]) and dense, high biomass material, such as whale falls and other carrion (e.g. [4,5]). To consume episodic, localized, and often recalcitrant material, deep-sea taxa have developed an array of specialized adaptations. Well-studied organisms that capitalize on these food sources include the bone-eating polychaete *Osedax* [6], the wood-boring mollusc *Xylophaga* [7], and the many taxa that rely on chemosynthesis at hydrothermal vents and cold seeps (e.g. [8]). These trophic specialists forge important connections in global ocean food webs, contributing to energy and nutrient cycling (e.g. [9]).

Another alternative food source for deep-ocean fauna is plant and macroalgal debris. This material can reach abyssal (4000−6000 m) and hadal (6000−11 000 m) habitats and may serve as an important trophic link between the surface and organisms at great depths (e.g [10–13]). For example, the holopelagic macroalgae *Sargassum* spp. has been observed on the seafloor in and around the western Atlantic (e.g [10,11,14–16]). At the surface, the quantity and distribution of *Sargassum* have increased since 2011, expanding from the Sargasso Sea to the central Atlantic Ocean and Caribbean Sea (e.g. [17–19]), causing ecosystem and economic impacts on coastal waters through large beaching events [20]. While *Sargassum* could be a growing source of labile carbon to the deep ocean (e.g. [21,22]), the fate of this organic material and the organisms that consume it have not been fully investigated. This is in part due to the technical challenges of studying abyssal and hadal ecosystems, as most studies use untethered landers baited with carrion to collect video and physical samples from a single location (e.g. [23–25]).

In this study, we opportunistically encountered the swimming isopod *Bathyopsurus nybelini* Nordenstam, 1955 (Suborder Asellota, Family Munnopsidae Lilljeborg, 1864) carrying *Sargassum* at abyssal and hadal depths in the western Atlantic Ocean and Caribbean Sea. Previous lander-based pictures [15] and gut contents from trawled specimens collected in poor condition during the 1948 Swedish Deep-Sea Expedition to the Puerto Rico Trench [26,27] suggested this organism may consume *Sargassum*. We leveraged the newly refit Deep Submergence Vehicle (DSV) *Alvin*, which as of 2022 can now reach depths exceeding 6000 m [28,29], to explore the importance of surface-derived macroalgae on the lifestyle of *Bathyopsurus* using *in situ* video, morphological analysis, and DNA sequencing of the gut microbiome. We provide conclusive evidence that *Bathyopsurus nybelini* is specialized to feed on *Sargassum*, forming a trophic link between the surface and organisms at the deepest ocean depths across the tropical Atlantic and Caribbean Sea.

## 2. Results

### (a) Distribution of macroalgae and observation of isopods at abyssal and hadal depths

**Figure 1.**
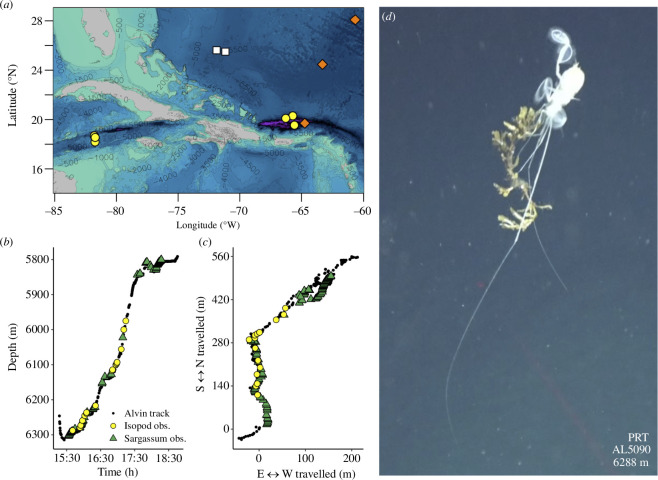
The swimming isopod *Bathyopsurus nybelini* was consistently observed carrying *Sargassum* at abyssal and hadal depths. (*a*) A map showing observations of *B. nybelini* during this study and historical records. Yellow circles, this study; orange diamonds, 1948 Swedish Deep-Sea Expedition collections [26,27]; white squares, lander observations [15]. (*b,c*) Isopod and macroalgae observations along the seafloor during *Alvin* dive AL5090 as a function of depth, time, and relative distance travelled. Data begin shortly before the sub arrives on the seafloor. The legend is the same for both panels. (*d*) Representative image of *B. nybelini* carrying *Sargassum*. PRT, Puerto Rico Trench.

We explored the Puerto Rico Trench, Mid-Cayman Spreading Center, and surrounding waters on 14 dives with DSV *Alvin*. Video from three dives in the Puerto Rico Trench (depths 5605−6303 m), each with 2–3 h of bottom time and covering <2 km, showed that macroalgae was abundant on the seafloor (figure 1, electronic supplementary material, figures S1 and S2). Macroalgae was observed >300 times on each dive at an average interval of less than 4 m between observations. Much of this material was visually identifiable as *Sargassum*. Falls ranged in size from small pieces to large piles >50 cm in length and were commonly identified on soft sediment. Macroalgae was seen floating past the submersible while being carried by the current and often became entangled around benthic organisms, including glass sponges and anemones (electronic supplementary material, figure S1). Hundreds of macroalgal fragments <3 cm were observed when the camera zoomed in on the seafloor but were not quantified.

Swimming isopods were consistently observed interacting with *Sargassum* (electronic supplementary material, videos S1–S4). Thirty-two distinct individuals were seen at six locations (depths 5002−6288 m), three within the Puerto Rico Trench (Central Ridge, North Wall, and South Wall) and three near the Mid-Cayman Spreading Center (Axial Volcanic Ridge, North Ridge, and Beebe Woods; figure 1, table 1, electronic supplementary material, table S1). Nineteen individuals were filmed carrying *Sargassum*; in some cases, the frond was longer than the body length of the isopod. Isopods swam by paddle stroking above the seafloor with their two natatory legs (pereopods V–VI). Individuals swam backwards, and in some instances upside down, either travelling parallel to or vertically away from the seafloor. Isopods swam continuously, with a mean frequency of 1.27 ± 0.38 strokes s^−1^ (range 0.60–2.15 strokes s^−1^). The isopods carried *Sargassum* off the seafloor by pinching gnathopod 1.

**Table 1.** Observations of *Bathyopsurus nybelini* in the Puerto Rico Trench and Mid-Cayman Spreading Center on the *Alvin* Science Verification Expedition (AT50−02). Date and encounter time are reported in GMT. Individuals that were observed carrying *Sargassum* are indicated with a Y (yes), while individuals that were not carrying *Sargassum* are shown with an N (no). Observations where it was not possible to determine if the isopod was carrying *Sargassum* due to distance or focus are blank. Individual 5089–05, denoted by an asterisk, was seen six times over a short period, with the initial encounter time and location reported here. Example videos and paddle stroke frequencies are available in the electronic supplementary material.

Location	*Alvin* dive ID	Encounter ID	Dive site	Date	Encounter time (GMT)	Latitude (°N)	Longitude (°W)	Depth (m)	Carrying *Sargassum*
Puerto Rico Trench	5089	5089-01	Central Ridge	7.29.22	16:38:46	19.53967	−65.57386	6103.5	N
Puerto Rico Trench	5089	5089-02	Central Ridge	7.29.22	16:47:22	19.54050	−65.57327	6095.1	Y
Puerto Rico Trench	5089	5089-03	Central Ridge	7.29.22	16:47:40	19.54054	−65.57324	6094.4	N
Puerto Rico Trench	5089	5089-04	Central Ridge	7.29.22	16:59:19	19.54188	−65.57231	6069.3	Y
Puerto Rico Trench	5089	5089-05*	Central Ridge	7.29.22	17:01:12	19.54194	−65.57227	6067.6	N
Puerto Rico Trench	5089	5089-06	Central Ridge	7.29.22	17:27:08	19.54355	−65.57149	6034.8	N
Puerto Rico Trench	5090	5090-01	North Wall	7.30.22	15:40:32	20.29516	−65.70579	6287.6	Y
Puerto Rico Trench	5090	5090-02	North Wall	7.30.22	15:53:31	20.29542	−65.70582	6279.2	N
Puerto Rico Trench	5090	5090-03	North Wall	7.30.22	15:54:56	20.29549	−65.70579	6273.1	Y
Puerto Rico Trench	5090	5090-04	North Wall	7.30.22	15:58:01	20.29576	−65.70579	6259.2	Y
Puerto Rico Trench	5090	5090-05	North Wall	7.30.22	16:01:55	20.29596	−65.70572	6245.3	Y
Puerto Rico Trench	5090	5090-06	North Wall	7.30.22	16:04:55	20.29614	−65.70576	6238.9	
Puerto Rico Trench	5090	5090-07	North Wall	7.30.22	16:05:09	20.29617	−65.70576	6236.3	Y
Puerto Rico Trench	5090	5090-08	North Wall	7.30.22	16:20:55	20.29653	−65.70581	6216.8	
Puerto Rico Trench	5090	5090-09	North Wall	7.30.22	16:50:23	20.29683	−65.70581	6117.0	Y
Puerto Rico Trench	5090	5090-10	North Wall	7.30.22	16:51:03	20.29678	−65.70593	6114.3	Y
Puerto Rico Trench	5090	5090-11	North Wall	7.30.22	16:56:49	20.29691	−65.70581	6100.2	Y
Puerto Rico Trench	5090	5090-12	North Wall	7.30.22	16:58:15	20.29695	−65.70576	6096.8	Y
Puerto Rico Trench	5090	5090-13	North Wall	7.30.22	16:59:02	20.29699	−65.70571	6093.1	Y
Puerto Rico Trench	5090	5090-14	North Wall	7.30.22	16:59:02	20.29699	−65.70571	6093.1	
Puerto Rico Trench	5090	5090-15	North Wall	7.30.22	16:59:02	20.29699	−65.70571	6093.1	
Puerto Rico Trench	5090	5090-16	North Wall	7.30.22	17:06:22	20.29735	−65.70536	6055.9	Y
Puerto Rico Trench	5090	5090-17	North Wall	7.30.22	17:10:51	20.29750	−65.70520	5999.8	Y
Puerto Rico Trench	5090	5090-18	North Wall	7.30.22	17:14:25	20.29769	−65.70516	5974.9	Y
Puerto Rico Trench	5091	5091-01	South Wall	7.31.22	17:55:01	20.09438	−66.27648	5868.6	Y
Puerto Rico Trench	5091	5091-02	South Wall	7.31.22	18:38:42	20.09536	−66.27606	5806.2	
Puerto Rico Trench	5091	5091-03	South Wall	7.31.22	18:45:27	20.09584	−66.27616	5753.9	Y
Mid-Cayman Spreading Center	5094	5094-01	Axial volcanic Ridge	8.8.22	17:48:23	18.17784	−81.72858	5191.7	Y
Mid-Cayman Spreading Center	5097	5097-01	North Ridge	8.11.22	16:19:13	18.68616	−81.78744	6026.5	N
Mid-Cayman Spreading Center	5097	5097-02	North Ridge	8.11.22	16:25:55	18.68445	−81.78717	6040.5	Y
Mid-Cayman Spreading Center	5101	5101-01	Beebe Woods	8.15.22	18:10:49	18.54712	−81.71882	5001.9	N
Mid-Cayman Spreading Center	5101	5101-02	Beebe Woods	8.15.22	18:10:57	18.54713	−81.71881	5001.9	N

### (b) Integrative taxonomy of *Bathyopsurus nybelini*

**Figure 2.**
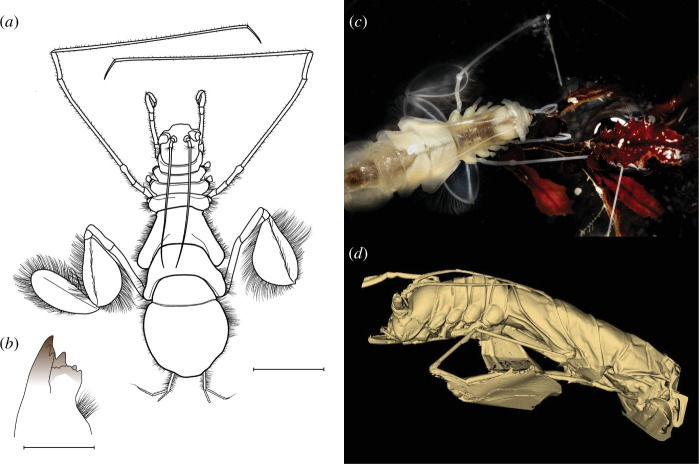
*Bathyopsurus nybelini* is morphologically adapted to feed on *Sargassum* using large paddles for a specialized swimming stroke and mouthparts for tearing. (*a*) Whole body dorsal illustration of collected female specimen with 2 cm scale. (*b*) Left mandible with 0.5 mm scale. Credit: Johanna Weston. (*c*) Isopod staged with associated *Sargassum*. Credit: Daniel Hentz. (*d*) Micro-CT scan showing lateral morphology at 19.4 µm resolution. Credit: Mackenzie Gerringer. All images are specimen AT50−02−017.

Two isopods were collected from depths of 6100 and 6114 m within the Puerto Rico Trench (Dive AL5090; figure 2, table 1). Specimens are female, with total body lengths of 33.6 and 35.2 mm. Their bodies are transparent and thin, of parchment-like consistency, with highly fragile appendages. We identify these individuals as *Bathyopsurus nybelini* due to (i) the separation of the first four pereion segments, of equal length and width, from the expanded and evenly rounded pleotelson, (ii) the lack of molar process and palp on the mandibles, (iii) a left mandible having five teeth on the incisor, and (iv) the operculum being broader than long with near parallel front and hind margins. This new material informed a redescription of characters that were absent or damaged during previous trawl-based collections (electronic supplementary material; [26,27]). Ribosomal 18S and 28S RNA gene sequencing placed *B. nybelini* within the Bathyopsurinae sub-family and sister to *Paropsurus giganteus* Wolff, 1962 [27] (electronic supplementary material, figures S3 and S4).

Both individuals were carrying *Sargassum* at the time of collection. The macroalgae was morphologically identified as *Sargassum natans* VIII (electronic supplementary material; [30]). Fronds hosted an epiphyte community that covered <20% of the surface area. Epibiont species present included the hydroid *Aglaophenia latecarinata*, the polychaete *Spirorbis spirorbis*, and a filamentous alga, suggesting rapid deposition from the surface and a lack of degradation (electronic supplementary material). *Sargassum* was nitrogen poor. Carbon (C) and nitrogen (N) reflected 28.3% and 0.59% of the biomass dry weight, respectively, yielding a C:N molar ratio of 56.3.

The gross morphology from both direct examination and micro-CT scanning revealed key functional adaptations to support the isopod’s feeding on macroalgae (figure 2). The specimens appear to lack eyes; the long flagella of the antennule and sensory setae may allow the isopod to find macroalgae on the seafloor by touch or chemosensation. Individuals carried *Sargassum* by compressing the dactylus and propodus on the carpus in pereopod I (electronic supplementary material, figure S5). The serrated pereopod I dactylus and mandible incisor and robust and knotty left lacinia mobilis aid in the tearing and grinding of *Sargassum* (electronic supplementary material, figure S6). The large dual paddles support the isopod’s characteristic swimming gait, which involves alternating power strokes of adjacent appendages. In addition to the broadening and separating of the two paddles on each appendage during the power stroke, setae provide additional surface area for propulsion power to carry large fronds of *Sargassum*. These paddles were missing in the original collections [26,27].

### (c) Gut content metagenomic sequencing

**Figure 3.**
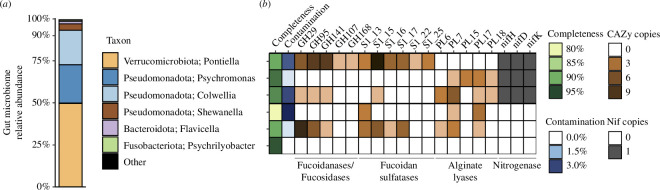
The gut microbiome of *Bathyopsurus nybelini* can degrade sulfated polysaccharides and fix nitrogen. (*a*) The composition of the gut microbiome based on the averaged relative abundances of ten single-copy marker genes. (*b*) Genome completeness, contamination, and gene copy numbers from gut metagenome-assembled genomes. The rows in (*b*) are aligned with their corresponding taxa in (*a*). CAZy, carbohydrate-active enzymes; Nif, nitrogenase genes.

Shotgun metagenomic sequencing of the isopod’s gut contents indicated a diet containing macroalgae. At least 30 marker genes related to *Sargassum* were present within the gut, including rRNA genes, cytochrome c oxidases, and ribosomal proteins (electronic supplementary material, figure S7). Most of these genes were identical to published sequences belonging to *Sargassum fluitans* III. Phylogenetically informative single-copy marker genes revealed the gut microbiome is composed primarily (>90%) of the genera *Pontiella* (phylum Verrucomicrobiota in GTDB-Tk and Kiritimatiellota in NCBI; electronic supplementary material, figure S8), *Psychromonas*, and *Colwellia* (figure 3). Members of the genera *Shewanella*, *Flavicella*, and *Psychrilyobacter* together represented approximately 6% of the remaining community. To determine if these microorganisms contribute to the digestion of macroalgal polysaccharides, we explored their genomic functional potential using metagenome-assembled genome (MAG) binning (electronic supplementary material, table S2). The MAGs within the gut belonging to *Pontiella* and *Flavicella* have putative genes for the degradation of fucose-containing sulfated fucoidans and polysaccharides, including fucosidases (GH29, GH95, GH141), fucanoidases (GH107, GH168), and fucoidan sulfatases (S1_13, S1_15, S1_16, S1_17, S1_22, S1_25; e.g. [31,32]). MAGs related to *Psychromonas*, *Colwellia*, and *Shewanella* have genes for the use of alginate (PL6, PL7, PL15, PL17, PL18; e.g. [33,34]). The three most abundant organisms in the gut—*Pontiella*, *Psychromonas*, and *Colwellia*—all contain the nitrogenase nif operon for N_2_ fixation, suggesting these organisms may provide the host with bioavailable nitrogen. While the *Psychromonas* and *Colwellia* MAGs are related to other deep-ocean lineages (electronic supplementary material, figures S9 and S10), the ability to fix nitrogen is absent in other deep-sea members of these genera (electronic supplementary material, figure S11).

## 3. Discussion

We provide conclusive evidence that the isopod *Bathyopsurus nybelini* is adapted to feed on surface-derived macroalgae in abyssal and hadal ecosystems of the Atlantic Ocean and Caribbean Sea using a specialized swimming style, serrated mouthparts, and a diazotrophic gut microbiome. Our work shows that *Sargassum* is a direct trophic link between the surface and deep ocean and that organisms at great depths are using this material. The isopod appears to lack eyes, likely relying instead on specialized antennule and sensory setae to chemosense macroalgal odours (e.g. [35]). Video and morphological evidence highlight that this isopod has a specialized method of locomotion—swimming upside down and backward with large paddles—that allows it to carry *Sargassum* off the seafloor. Munnopsidae are commonly observed swimming in the deep ocean (e.g. [36–38]), although many swim only in short bursts to avoid predation (e.g. [39–41]). We hypothesize that *Bathyopsurus* may carry *Sargassum* into the water column to avoid predation at the seafloor. Future work to determine where this organism carries this algal material would reveal further details on the evolutionary significance of this behaviour. Together, our findings demonstrate that *Bathyopsurus* is specifically adapted to use a rather unconventional source of food at abyssal depths, illustrating the importance of food falls to deep-sea specialists.

In addition to the isopod’s morphological adaptations, we show that *Bathyopsurus* has a unique gut microbiome specialized in the degradation of macroalgal material. Brown algal cell walls are composed of sulfated fucoidans and alginates, polysaccharides that are recalcitrant and require highly specialized microorganisms for degradation (e.g. [31,42,43]). The high abundances of *Pontiella* that contain genes for the degradation of sulfated fucoidans (e.g. [44–46]), along with *Psychromonas* and *Colwellia* that can degrade alginate (e.g. [46,47]), are consistent with the isopod consuming macroalgae. These findings also agree with hypotheses from terrestrial isopods suggesting that gut microorganisms play an important role in host nutrition (e.g. [48,49]). While members of the genera *Psychromonas* and *Colwellia* are commonly reported on sinking particles in the deep sea [50] and within the gut microbiomes of hadal animals (e.g. [51–53]), the presence of *Pontiella* here reveals a distinction from the gut microbiomes of other deep-ocean organisms that feed on carrion, which instead have high abundances of Mycoplasmataceae (e.g. [53,54]). All three of these MAGs reported here are capable of fixing N_2_ gas into biologically available N, a finding not previously observed in microbiomes of abyssal and hadal animals. Because *Sargassum* is a carbon-rich, stoichiometrically imbalanced food source with a C:N ratio above 20:1 (our data; e.g. [18,22,55]), N_2_ fixation may provide a source of supplemental N for both the microorganism and host. The association of diazotrophs with animals occurs across many marine and terrestrial symbioses (e.g. [56,57]), especially those which feed on carbon-rich substrates, including urchins [58], wood-boring bivalves [59,60], and insects [61–63]. Therefore, our findings support the hypothesis that microorganisms form an important trophic link between detritus and larger organisms at great depth (e.g. [11,22,64,65]), with herbivorous detritivores supported by a distinct gut microbiome capable of degrading algal carbon-rich polysaccharides and supplementing N content via N_2_ fixation. Our results provide a unique example of how some deep-sea megafauna rely on specialized microbial communities to access varied nutrient and energy sources. While the importance of chemosynthetic microorganisms to higher trophic levels has been well documented (e.g. [66]), a complete picture of global ocean food webs requires interdisciplinary investigations that integrate across taxa and scales to understand these vital connections.

From a biogeographic perspective, our observations in both the Atlantic and Caribbean at distances more than 1500 km apart indicate this isopod species is widely distributed and consuming *Sargassum* at depths of 5000−7900 m across the *Sargassum* belt. *Bathyopsurus nybelini* has also been reported from the abyssal plains near the Kermadec Trench in the Southwestern Pacific and Tasman Sea (depths 4400−5900 m, [27]), albeit without molecular identification, and the genus was found in the Southern Ocean [67]. Isopods are among the most abundant and diverse fauna in the deep ocean [68]. Both shallow- and deep-sea isopods have been observed associated with macroalgae [11,69–71], although many species are generalists and detritivores, consuming a wide range of food sources (e.g. [39,72,73]). Previous analyses of *B. nybelini* gut contents documented both macroalgae and rare remnants of other organisms [27], suggesting macroalgae may not be its only food source. Episodic pulses, seasonal cycles, and climatic changes in primary production can impact organismal distributions and physiology in the deep ocean (e.g. [74,75]). Given that *Sargassum* abundances at the surface can show seasonality, patchy distributions, and have been increasing over the last decade (e.g. [16]), future work should link *B. nybelini* distributions and behaviour to estimates of *Sargassum* biomass at the surface and its depositional flux using satellites, sediment traps, and transects along the seafloor. Overall, we hypothesize that *Bathyopsurus* and related herbivorous isopods may be prominent members of benthopelagic megafauna communities across the world’s oceans in areas with macroalgal deposition.

Our finding that surface-derived macroalgae is common at the abyssal-hadal boundary and actively eaten by deep-ocean taxa may have implications for carbon cycling and storage at great depth. Exposure of macroalgae to turbulence and deep-mixing events that exceed depths of 130 m (1.3 Megapascals), which can occur during storms [76,77], are enough to make *Sargassum* negatively buoyant and rapidly sink to the abyssal seafloor in approximately 40 h (sinking speeds of approx. 3000 m d^−1^ [78]). The many *Sargassum* observations here suggest this fixed carbon source is readily available and may be abundant in the abyssal ocean (e.g. [15,21,22]). On a global scale, total carbon sequestration from macroalgae is estimated to be about 173 Tg C yr^−1^, 88% of which is in the deep oceans [21]. The fate of *Sargassum* is important as its abundance and distribution in the tropical Atlantic and Gulf of Mexico have been increasing, with important ramifications for both the ecology and economies in the Atlantic (e.g. [20,79,80]). Furthermore, it has been suggested to combat climate change by deliberately sinking macroalgae into the deep sea to store carbon (e.g. [81,82]). *Bathyopsurus nybelini* is not the only species that consumes macroalgae; pulses of organic matter can elicit responses by sea cucumbers, crustaceans, polychaetes, cumaceans, and microorganisms (e.g. [11,12,70,83–86]). Given that numerous organisms consume this material, increasing biomass flux to the seafloor will have downstream impacts of largely unknown consequence on the ecology of deep-sea communities (e.g. [87,88]). Future work will be needed to quantitatively evaluate the flux of *Sargassum* and its relative importance to the deep-ocean food web.

While interest in the deep ocean has increased over the last few decades [89], sampling at abyssal and hadal depths remains constrained by technology that is limited to only a few nations and institutions [90]. Following its upgrade, DSV *Alvin* is now one of only a few human-occupied vehicles capable of reaching depths exceeding 6000 m (e.g. [91,92]), providing increased opportunity to actively image and sample the deep abyssal and hadal seafloor. Our observations using *Alvin* follow previous lander-based images [15] and gut content morphological analysis of trawled specimens [27] that suggested this organism consumes *Sargassum*. Our study highlights how the ability to selectively target and collect specimens from the deep sea fills gaps in knowledge about the fate of fixed carbon and nutrients in surface waters and uncovers novel adaptations that link oceanic food webs. The deep ocean—the largest biome on the planet—is commonly deemed ‘extreme.’ We add to a growing body of evidence that abyssal and hadal communities are directly impacted by conditions at the surface, including through the deposition of pollutants and plastics (e.g. [93–95]). Further exploration will only continue to reveal that the fate of organisms at the greatest ocean depths is inexorably connected to human activities far above.

## 4. Methods

### (a) Study location and methodology

Samples were collected aboard the R/V *Atlantis* during the *Alvin* Science Verification Expedition (AT50−02) to the Puerto Rico Trench (26 July–2 August 2022) and the Mid-Cayman Spreading Center (3−19 August 2022). Video and sampling were performed using *Alvin* during five scientific dives surrounding the Puerto Rico Trench (AL5088−5092; depths approx. 5600–6300 m) and nine dives at the Mid-Cayman Spreading Center (AL5093−5101; depths approx. 2300–6000 m). Average bottom time for each dive was 3 h. *Alvin* was equipped with two 4K ultra-high-definition Optim SeaCam cameras (Deep-Sea Power and Light, San Diego, CA) on pan and tilt platforms. Lighting was provided by ten LSL-2000 SeaLights (Deep-Sea Power and Light).

### (b) Estimating *Bathyopsurus* and macroalgae abundances on the seafloor

The isopod *Bathyopsurus nybelini* was encountered during six dives (table 1). Macroalgae pieces bigger than approximately 3 cm were quantified during three dives (dives AL5089, AL5090, AL5091) within the Puerto Rico Trench along sedimented seafloor where isopods were also observed. Dives had multiple objectives, including verification of navigation systems, collections, and other protocols; therefore, straight transects were not typically conducted. Due to the lack of fixed transects and non-calibrated laser scale indicators, quantitative abundances of isopods and macroalgae were not determined. Instead, we report the total number of observations on each dive relative to the duration of bottom time and total distance travelled. Observations were plotted using the R [96] packages marmap [97] and rgdal v. 1.5-28.

### (c) Sample collection

Two individual isopod specimens carrying *Sargassum* were collected via a suction sampler into the same chamber, approximately four minutes apart, from 6114 and 6100 m depth (dive AL5090; 20.29679°N, 65.70597°W and 20.29694°N, 65.70575°W). Upon return to the surface, the specimens were kept in cold seawater on ice and moved to a 4°C cold room for processing. One isopod (AT50-02-017) and one frond of *Sargassum* were preserved in 95% ethanol as vouchers for morphological taxonomy. The second isopod (AT50-02-018) was sampled for phylogenetic and microbiological analyses as described below and then preserved in 95% ethanol. The second piece of *Sargassum* was subsampled for chemical content. All sub-samples were flash-frozen in liquid N_2_ and stored at −80°C.

### (d) Isopod and *Sargassum* morphology

The collected isopod and *Sargassum* material were morphologically examined with a Zeiss Axio Zoom V16 stereo microscope (Oberkochen, Germany). Images were digitally inked using Inkscape v.1.2.2. For the isopod, terminology, measurements, and identifications follow Wolff, 1962 [27], Wilson, 1989 [98], and Osborn, 2009 [37]. Total body length was measured medially from the anterior edge of the cephalon to the posterior tip of the pleotelson. The length of segments was measured medially or laterally from the anterior margin to the posterior margin. For the *Sargassum*, terminology and morphotype determination follow Parr, 1939 [30] and Schell *et al*., 2015 [17]. Further details can be found in the electronic supplementary material.

### (e) 3D imaging using micro-computed tomography

One specimen (AT50-02-017) was imaged using micro-computed tomography (micro-CT) scanning using a Bruker SkyScan 1173 (Karel F. Liem Bioimaging Center, Friday Harbor Laboratories, University of Washington, USA). The isopod was wrapped in plastic with a minimal quantity of 75% ethanol to keep the specimen wet during scanning. Two scans were conducted at 50 kV and 145 µA, with no metal filter, optimized for low-density invertebrates. Voxel size was 19.4 µm resolution for the first, full body scan and 7.1 µm for the second, head-specific scan. After scanning, two-dimensional images were reconstructed using NRecon (Bruker, 2005−2011). During reconstruction, we followed a standard protocol for optimizing *x*/*y* alignment, reducing ring artefacts generated by variations in sensitivity on the scanner’s detector, correcting for beam hardening, and post-aligning the scan (e.g. [99]). To reduce scan size for analysis, the reconstructed scans were segmented in DataViewer (Bruker, 2004−2011). Images for this manuscript were rendered using the open-source image computing platform 3D Slicer via the extension SlicerMorph [100,101].

### (f) Isopod DNA barcoding

Total genomic DNA was extracted from one isopod (AT50-02-018) ventral pleotelson using a standard phenol-chloroform approach. Two regions for PCR amplification were targeted using primers previously used on the Munnopsidae [37]: approximately 1800 bp of the nuclear small-subunit 18S rRNA gene with mitchA and mitchB [102] and approximately 1100 bp surrounding the D1−D3 region of the nuclear large-subunit 28S rRNA gene with LSUD1F and D3AR [103]. PCR products were purified using a QIAquick PCR Purification kit (Qiagen) and sequenced by Sanger sequencing on an ABI 3730XL capillary sequencer (Eurofins Genomics, Louisville, KY, USA). Electropherograms were manually inspected, and ambiguous base calls were denoted with N. Primers were trimmed in MEGA X [104]. Phylogenetic analyses are described in the electronic supplementary material.

### (g) *Sargassum* biomass carbon and nitrogen content

Approximately 1 g of flash-frozen *Sargassum*, which was actively carried by an isopod during collection, was stored at −80°C. This material included epiphytic polychaetes that were not removed prior to analysis as we have no evidence they are selectively removed by the isopod prior to consumption. *Sargassum* was freeze-dried, homogenized, and subsampled. Carbon and nitrogen content were determined using an Exeter Analytical CE-440. The %C and %N data were used to calculate molar C:N ratios.

### (h) Gut content metagenomic sequencing

For gut content metagenomic shotgun sequencing, gut contents were aseptically obtained from one isopod (AT50-02-018) by placing a syringe needle through the abdomen wall. DNA was extracted using a Qiagen DNeasy Blood and Tissue Kit (Hilden, Germany) and shotgun metagenomic sequencing was performed on an Illumina Novaseq (Novogene, Sacramento, CA). Raw reads were quality trimmed using Trimmomatic v. 0.39 [105] and assembled with MEGAHIT v. 1.2.9 [106]. The depth of coverage of contigs was estimated using Bowtie 2 v. 2.3.5.1 [107] and SAMtools v. 1.10 [108].

We characterized the microbial community and its functional potential. Open reading frames were identified with Prodigal v. 2.6 [109] and Prokka v. 1.14.6 [110]. Gene annotation and the identification of carbohydrate active enzymes and sulfatases were performed using GhostKOALA [111], dbCAN3 [112] and SulfAtlas [113], respectively. Ten microbial single-copy marker genes (*rps2*, *rps7*, *rps8*, *rps15*, *rpl3*, *rpl5*, *rpl16*, *rpoB*, *recA*, 16S rRNA) were used to characterize the prokaryotic community based on their depth of coverage. Single-copy marker genes related to *Sargassum* were identified by blastp [114] comparisons against published *Sargassum* mitochondrial genomes. Trees were created by sequence alignment with MUSCLE [115], built using FastTree [116] and visualized using iTOL [117].

Metagenome-assembled genomes (MAGs) were obtained using MetaBAT 2 v. 2.11.1 [118]. Contigs longer than 5 kb were retained. The quality of each MAG was evaluated using CheckM v. 1.0.13 [119]. MAGs related to *Colwellia* and *Psychromonas* were further refined manually, including through blastn similarity against published genomes of these genera. Genomes were taxonomically classified with GTDB-Tk v. 1.6.0 [120] using Kbase [121]. Average nucleotide identity (ANI) comparisons were performed using OrthoANI [122]. Whole-genome trees of selected MAGs were created using concatenated single-copy marker genes obtained and aligned using CheckM, constructed using FastTree, and visualized using iTOL. Further comparative analyses can be found in the electronic supplementary material.

## Data Availability

Isopod specimens have been deposited at the Smithsonian Institution National Museum of Natural History (Suitland, MD, USA) under accession numbers USNM 1716401 and 1716402. CT-scan reconstructions are available on MorphoSource (Duke University) under accession number 000640894. Isopod and *A. latecarinata* rRNA gene sequences are available at NCBI under accession numbers PP351657, PP351679, and PP351649. The gut metagenome and MAGs are available at NCBI BioProject accession number PRJNA1075769. Raw video data are available from the Woods Hole Oceanographic Institution. Supplementary material is available online [123].
